# Elements of Practical Medicine

**Published:** 1885-07

**Authors:** 


**Elements of Practical Medicine.** By Alfred H. Carter, M. D., Physician to Queen's Hospital, &c. Third edition. New York: D. Appleton & Co., No 1 Bond street. 1885.

We are glad to welcome a new edition of this excellent book. While not to be compared with the larger standard works on practice, it serves admirably as a general introduction to the study of medicine, and is, therefore, well adapted for the student. At the same time, the practitioner who has not the leisure to read the larger and more complete works, will find here the essentials of the subject conveniently condensed and arranged for the refreshing of the memory. This new edition is excellently printed and bound.

